# Correction: A sequential model of the contribution of preschool fluid and crystallized cognitive abilities to later school achievement

**DOI:** 10.1371/journal.pone.0315523

**Published:** 2024-12-05

**Authors:** Philippe Carpentier, Geneviève Morneau-Vaillancourt, Sophie Aubé, Célia Matte-Gagné, Anne-Sophie Denault, Mara Brendgen, Simon Larose, Amélie Petitclerc, Isabelle Ouellet-Morin, René Carbonneau, Bei Feng, Jean Séguin, Sylvana Côté, Frank Vitaro, Richard E. Tremblay, Ginette Dionne, Michel Boivin

There are errors in the Author Contributions. The correct contributions are:

**Conceptualization:** Philippe Carpentier, Célia Matte-Gagné, Anne-Sophie Denault, Simon Larose Amélie Petitclerc, René Carbonneau, Jean Séguin, Ginette Dionne, Michel Boivin.

**Formal analysis:** Philippe Carpentier, Geneviève Morneau-Vaillancourt, Sophie Aubé, Bei Feng.

**Funding acquisition:** Mara Brendgen, Isabelle Ouellet-Morin, Sylvana Côté, Frank Vitaro, Richard E. Tremblay, Ginette Dionne, Michel Boivin.

**Investigation:** Philippe Carpentier, Mara Brendgen, Sylvana Côté, Frank Vitaro, Richard E. Tremblay, Ginette Dionne, Michel Boivin.

**Methodology:** Philippe Carpentier, Bei Feng, Michel Boivin.

**Project administration:** Mara Brendgen, Amélie Petitclerc, Isabelle Ouellet-Morin, Sylvana Côté, Frank Vitaro, Richard E. Tremblay, Ginette Dionne, Michel Boivin.

**Resources**: Simon Larose, Ginette Dionne, Michel Boivin.

**Software:** Bei Feng

**Supervision:** Ginette Dionne, Michel Boivin.

**Writing–original draft:** Philippe Carpentier, Michel Boivin.

**Writing–review & editing:** Geneviève Morneau-Vaillancourt, Sophie Aubé, Célia Matte-Gagné, Anne-Sophie Denault, Mara Brendgen, Simon Larose, Amélie Petitclerc, Isabelle Ouellet-Morin, René Carbonneau, Bei Feng, Jean Séguin, Sylvana Côté, Frank Vitaro,

Richard E. Tremblay, Ginette Dionne, Michel Boivin
