# Supplementary material for: A sequential model of the contribution of preschool fluid and crystallized cognitive abilities to later school achievement
Source: PLoS One. 2022 Nov 18;17(11):e0276532. doi: 10.1371/journal.pone.0276532 (PMC9674147; doi:10.1371/journal.pone.0276532)
Supplement: S1 Table — (DOCX) [file pone.0276532.s001.docx]

| **Table S1. Missing value patterns and variations in model variable scores.** | | | | | | | | |
| --- | --- | --- | --- | --- | --- | --- | --- | --- |
|  | QNTS | | | | QLSCD | | | |
|  | No missing | 1-2 missings | >2 missings |  | No missing | 1-2 missings | >2 missings |  |
|  | N=194 (18.1%) | N=440 (41.0%) | N=438 (40.9%) |  | N=277 (17.5%) | N=620 (39.2%) | N=686 (43.3%) |  |
|  | M (SD) | M (SD) | M (SD) | *p value* | M (SD) | M (SD) | M (SD) | *p value* |
| ***Fluid abilities*** | | | | | | | | |
| Block Design 41M | - | - | - | - | 6.77 (3.62) | 6.28 (3.96) | 6.08 (3.76) | .05 |
| Block Design 63/73M | 20.89 (6.20) | 19.72 (6.52) | 18.98 (6.94) | .01 | 21.22 (9.36) | 19.78 (9.95) | 19.14 (10.17) | .04 |
| DCCS | 24.58 (7.41) | 24.24 (7.25) | 23.61 (7.27) | n.s. | - | - |  | - |
| VCR 41M | - | - |  | - | 3.57 (2.32) | 3.25 (2.19) | 3.09 (2.14) | .01 |
| VCR 63/73M | 5.17 (2.30) | 4.89 (2.21) | 4.65 (2.13) | n.s. | 6.03 (2.37) | 5.96 (2.41) | 5.71 (2.23) | n.s. |
| ***Crystallized abilities*** | | | | | | | | |
| Lollipop | 44.01 (11.38) | 42.42 (12.86) | 42.01 (14.63) | n.s. | 59.47 (5.16) | 57.96 (6.63) | 55.74 (9.17) | .001 |
| PPVT | 58.13 (18.54) | 53.78 (17.99) | 52.47 (20.42) | .01 | 84.79 (14.06) | 80.26 (16.67) | 76.66 (20.57) | .001 |
| NKT | 8.02 (4.33) | 7.69 (4.03) | 7.90 (4.28) | n.s. | 13.73 (2.93) | 13.23 (3.23) | 13.12 (3.65) | n.s. |
| ***School achievement*** | | | | | | | | |
| SA1 | 3.49 (1.05) | 3.25 (1.03) | 3.06 (1.11) | .001 | 3.89 (.96) | 3.68 (1.10) | 3.57 (1.12) | .001 |
| SA2/SA3 | 3.30 (1.02) | 3.11 (1.01) | 2.91 (1.17) | .001 | 3.88 (.93) | 3.62 (1.09) | 3.59 (1.09) | .001 |
| SA4 | 3.36 (1.09) | 2.99 (1.01) | 2.85 (1.17) | .001 | 3.69 (.96) | 3.55 (1.07) | 3.56 (1.11) | n.s. |
| SA6 | 3.46 (1.05) | 3.16 (1.10) | 3.20 (1.12) | .01 | 3.69 (1.07) | 3.54 (1.03) | 3.46 (1.07) | .03 |
| National exams | - | - | - | - | 73.99 (13.07) | 72.59 (13.87) | 70.81 (13.79) | .02 |
| ***Control Variable*** | | | | | | | | |
| **Mother Education** |  |  |  | n.s. |  |  |  | .001 |
| No diploma | 11.3% | 16.8% | 20.1% |  | 9.7% | 17.4% | 19.7% |  |
| Secondary diploma | 32.0% | 34.1% | 30.9% |  | 22.0% | 27.1% | 25.5% |  |
| Postsecondary diploma | 19.1% | 20.5% | 19.0% |  | 30.0% | 27.1% | 29.9% |  |
| University diploma | 37.6% | 28.6% | 30.0% |  | 38.3% | 28.4% | 24.8% |  |
| **Sex** |  |  |  | .01 |  |  |  | .01 |
| Male | 44.3% | 48.2% | 56.2% |  | 46.2% | 44.2% | 53.4% |  |
| Female | 55.7% | 51.8% | 43.8% |  | 53.8% | 55.8% | 46.6% |  |
| **Family Income** ^b^ | 7.49 (1.91) | 6.94 (2.21) | 6.70 (2.28) | .001 | 6.31 (2.06) | 6.01 (2.20) | 5.70 (2.32) | .001 |
| *Note.* Block Design and VCR were measured at 63 months in QNTS and at 41 months and 73 months in QLSCD; Grade 2 in QLSCD and grade 3 in QNTS; M = mean; *SD* = standard deviation; DCCS = Dimensional Change Card Sort; VCR = Visually Cued Recall Task; PPVT = Peabody Picture Vocabulary Test – Third Edition; NKT = Number Knowledge Test; n.s. = not significant.  ^b^ = 11 categories: (1) no income; (2) less than 5,000 CAD; (3) more or equal to 5,000 but less than 10,000 CAD; (4) more or equal to 10,000 but less than 15,000 CAD; (5) more or equal to 15,000 but less than 20,000 CAD; (6) more or equal to 20,000 but less than 30,000 CAD; (7) more or equal to 30,000 but less than 40,000 CAD; (8) more or equal to 40,000 but less than 50,000 CAD; (9) more or equal to 50,000 but less than 60,000 CAD; (10) more or equal to 60,000 but less than 80,000 CAD; (11) more or equal to 80,000 CAD. | | | | | | | | |
