# Supplementary material for: A sequential model of the contribution of preschool fluid and crystallized cognitive abilities to later school achievement
Source: PLoS One. 2022 Nov 18;17(11):e0276532. doi: 10.1371/journal.pone.0276532 (PMC9674147; doi:10.1371/journal.pone.0276532)
Supplement: S4 Fig — (DOCX) [file pone.0276532.s005.docx]

**Fig S4. Mediation Model of the Contributions of Preschool Fluid Abilities and Crystallized Abilities to School Achievement in the QLSCD (Two missing value condition).**

**
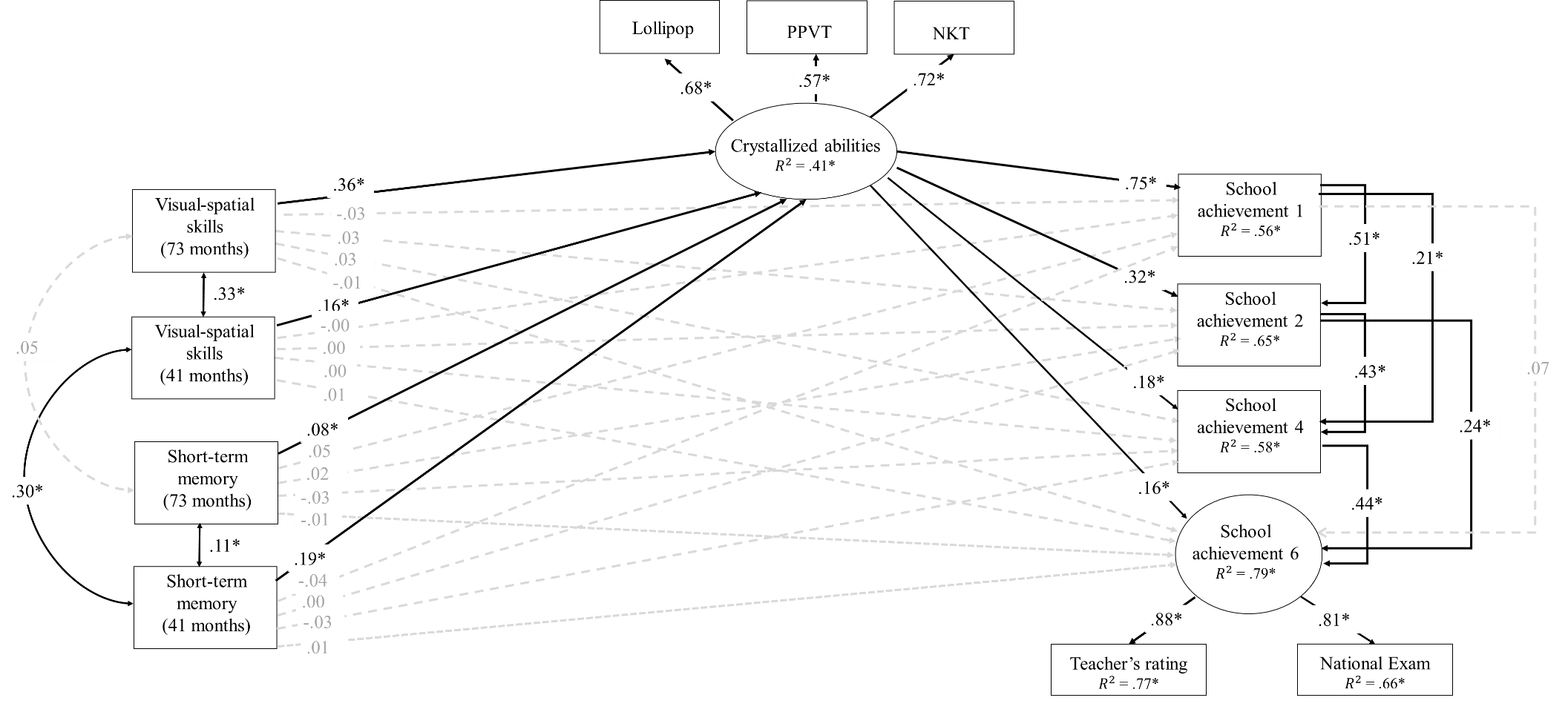
**

*Note.* χ^2^(37) = 158.57 *p* < .001; RMSEA = .06, 90% CI [.05, .07]; CFI = .97, TLI =.91. Nonsignificant contributions are indicated with dashed lines. Contributions of control variables (mother’s education, family income and sex) are not indicated to simplify the model. PPVT = Peabody Picture Vocabulary Scale, NKT = Number Knowledge test.
