# Supplementary material for: A sequential model of the contribution of preschool fluid and crystallized cognitive abilities to later school achievement
Source: PLoS One. 2022 Nov 18;17(11):e0276532. doi: 10.1371/journal.pone.0276532 (PMC9674147; doi:10.1371/journal.pone.0276532)
Supplement: S7 Fig — (DOCX) [file pone.0276532.s008.docx]

**Fig S7. Mediation Model of the Contributions of Preschool Crystallized Abilities and Fluid Abilities (Latent factor) to School Achievement in the QNTS (reverse model).**

*
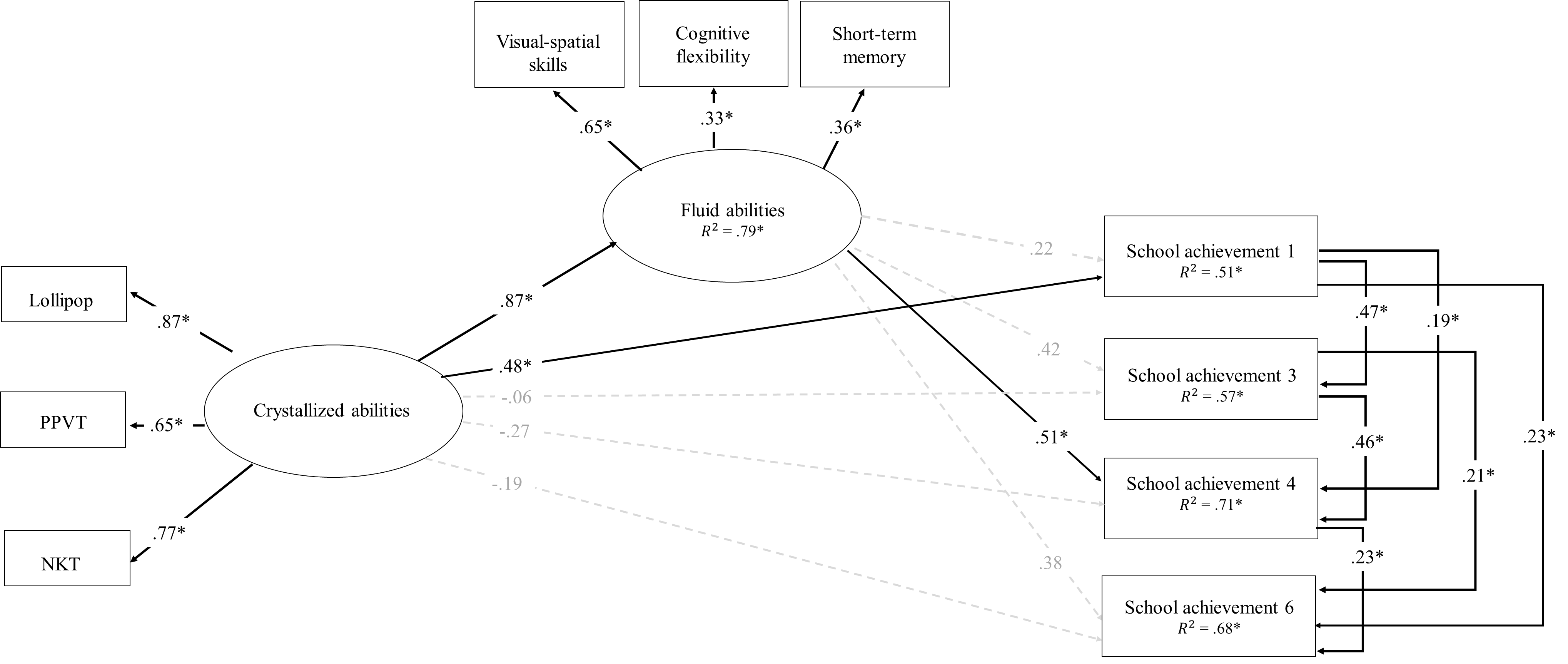
*

*Note.* χ^2^(36) = 93.72 *p* < .001; RMSEA = .04, 90% CI [.03, .05]; CFI = .98, TLI =.96. Nonsignificant contributions are indicated with dashed lines. Contributions of control variables (mother’s education, family income and sex) are not indicated to simplify the model. PPVT = Peabody Picture Vocabulary Scale, NKT = Number Knowledge test.
