# Supplementary material for: A sequential model of the contribution of preschool fluid and crystallized cognitive abilities to later school achievement
Source: PLoS One. 2022 Nov 18;17(11):e0276532. doi: 10.1371/journal.pone.0276532 (PMC9674147; doi:10.1371/journal.pone.0276532)
Supplement: Attachment — Submitted filename: Carpentier et al PloSOne Revision 1 Reviewers FINAL.docx [file pone.0276532.s010.docx]

Responses to the Editor’s comments

1. ***Did the authors consider potential confounds of (early measures of) IQ, such as socio-economic status or indices of input at home?***

As mentioned in the method section under subsection "Control Variables", all models controlled for maternal education (4-point scale from “high school not completed” to “university diploma obtained”), household income (11-point scale: from “less than 5000$” to “more than 80 000$”), and child sex (0, boys; 1 for girls). We now report the total unique contributions of these SES and sex variables in a new section where we present additional “Sensitivity analyses”. Specifically, we compared the variance accounted in the model when these controls were applied versus when they were not controlled for. The section now reads:

***Estimating the unique role of controls****. Finally, all models were tested controlling for mother education, family income and sex. To estimate the general unique contributions of these variables, we compared the variance accounted for in the model when these controls were applied versus not. In QNTS, the R^2^ was .45 (with controls) versus .39 (without controls) for Crystallized Abilities (latent score; 6% difference accounted for by the controls), and .67 (with controls) versus .65 (without controls) for School Achievement in Grade 6 (latent score; 2% difference accounted for by the controls), with very marginal changes in the predictive paths. In QLSCD, the R^2^ was .44 versus .38 for Crystallized Abilities (latent score; 6% difference accounted for by the controls), and .80 versus .77 for School Achievement in Grade 6 (latent score; 3% difference accounted for by the controls), again with very marginal changes in the predictive paths. Thus, even if these controls accounted for a significant part of the total variance in the models, the model still performed very well when these controls were applied.*

Thus, not only was the proposed mediational model not entirely accounted for by these SES and sex characteristics, but also it accounted for an impressive proportion of the variance in school achievement over and above these control factors. We now discuss these results considering Bailey et al, (2017; 2018) and Tricot and Sweller (2014) (see below).

2. ***The authors discuss stability of school achievement, but how about stability of IQ over time? Particularly at such a young age. Might time limited effects of IQ on school achievement also be understood in terms of instability of IQ for young children?***

As mentioned in the introduction letter, the block design (BD) and memory (MT) tasks were also administered at age 41 months in QLSCD (but not in QNTS, which explains why we did not consider the QLSCD 41-month data in the first version of the manuscript). The stability correlations between ages 41 and 73 months were r = .36 for the BD and r = .12 for the MT (see Table 2). So, yes, the stability was moderate at best for BD and quite low for the MT, especially when compared to the stability of school achievement. This difference in stability between measures of the two epochs may reflect developmental processes (i.e., more rapid developmental change in the early years), and likely the ad hoc nature of the assessments (i.e., BD and MT tasks are short and perhaps of limited reliability/stability with young children). Therefore, to account for this lack of stability and take advantage of this early information, we now include the 41-month assessments in the model. Doing so highlighted the early predictive value of these fluid abilities and the mediation model. That is, the prediction of the crystallized ability latent score was enhanced (to 44% of the variance compared to 37% in the original model, i.e., when only the 73-month scores were considered), with BD-41 (Beta = 17, p < .05) and MT-41 (Beta = .21, p < .05) now significantly adding to the prediction over and above the 73-month assessments. Interestingly, the unique contribution of crystallized abilities to grade 4 achievement is now significant (Beta = .15, p < .05), which confirms the extended developmental reach of these abilities throughout primary school (over the progressive auto-regressive prediction of school achievement). Again, the small unique prediction of BD-73 months to grade 2 achievement was significant. In other words, considering both BD and MT at 41 months in addition to BD and MT at 73 months significantly enhanced the predictive value of the mediation model and illustrate the importance of these early predictors despite their lack of stability in early development.

3. ***I wondered about the measures of crystallized abilities. To me, some of these tasks seem a rather direct measure of skills that are later considered school achievement. Could the relation, or rather difference, between (early) school achievement and crystallized abilities be explained a bit more?***

Indeed, general school achievement reflects children’s performance relative to their peers in topical domains that are central to the primary school curriculum (typically the “3 Rs”: reading, writing and arithmetic). There are thus, by definition, more advanced crystallized abilities. The main point is that these early crystallized abilities substantially predict how well children will do in primary school. This was acknowledged in the discussion. We have slightly expanded this section which now reads:

…However, *the diminished unique contributions of early crystallized abilities to school achievement beyond grade 3 does not mean that crystallized abilities are no longer relevant, but rather that they have become embedded in school achievement. Indeed, although they were not directly measured here, skills and knowledge acquired in school progressively becomes new, specific crystallized abilities which are reflected in children’s overall school achievement. The main point here is that children with strong crystallized abilities enter formal school with a head start, and then tend to keep this advantage over time. Furthermore, in the QLSCD the additive contribution of early crystallized to school achievement could be seen in the later grades, thus reflecting the long-term developmental reach of crystallized abilities.*

4. ***It is mentioned (page 12) that there were ceiling effects for some of the measures. How might this have affected the results? Was anything done about this?***
5. ***Have the authors considered the distributions of the variables included in the model and the potential effect thereof on the results?***

The distribution skewness of the main variables in QNTS was modest (ranging between -0.5 and 0.5), and therefore minimally affected by “floor/ceiling effects”. Only for QLSCD were the Lollipop and the NKT presenting a slight asymmetry mainly due to some modest ceiling effects. We used MLR (robust) under Mplus, which is robust to non-normality. It is likely that the calculation of a latent score of crystallized abilities minimized potential biases. Furthermore, there is reassurance in the strong convergence of findings supporting the model in the two cohorts. However, we now acknowledge this limitation in the discussion:

… *Third,* *although there was minimal asymmetry in the data, the Lollipop and the NKT presented a slight asymmetry mainly due to some modest ceiling effects. We used MLR (robust) under Mplus, which is robust to non-normality. It is also likely that the calculation of a latent score of crystallized abilities minimized potential biases.*

6. ***In line with comments of reviewer 1, I noticed that there is a significant amount of missing data. Does the model presented hold when only cases with complete data (at least on the most relevant measures) are considered?***

The missing data question is a challenging one as each of the two cohorts had its own missingness issues; missing data in QNTS was more pronounced for school achievement in grades 1 and 6, whereas in QLSCD there was more missing data in grades 4 and 6 (see Table 1). Missingness was also largely distributed across measures and participants and over time, such that only a minority of participants had complete data on all 15 measures (QLSCD; 13 for QNTS). Furthermore, it was important to minimize changes to the center piece of the model (i.e., mediation role of crystallized abilities) and the direct assessments of fluid abilities. Accordingly, we went for a compromise that allowed for a significant number of participants in the analyses, yet under stricter rules for inclusion. That is, in addition to the model (FIML) presented in the paper, we ran the model for participants who (1) had only one or two missing values, as well as for (2) participants with no missing data (i.e., listwise; only a minority of participants). The model withstood the test very well, with strong mediation again found in all models and most of the significant pathways confirmed. These analyses are presented in a new section “Sensitivity Analyses”, and the resulting model are now presented in Supplementary Figures S1 to S4. The section reads:

*Examining the role of missing data. Missing data was significant and largely distributed across the measures and participants over time. Missingness was non-random as indicated by Table S1 in the Supplementary material. To verify the robustness of the outcome model, we tested it under stricter rules of participant inclusion: one condition only involved participants having one or two missing values; another, with participants for which all measures were available (i.e., listwise). As shown in Supplementary Figures S1 to S4, the model withstood the test very well; strong mediation was again found in all models, and most of the significant pathways were confirmed, except for those of low magnitude in the listwise models who were not significant due to diminished power.*

In the discussion, we present this as evidence of the robustness of the model.

7. ***How well can fluid and crystallized abilities actually be distinguished in young children? Whereas the crystallized abilities seem interrelated, as expected, correlations among the fluid measures are much lower. In fact, all the fluid measures correlated more strongly with the crystallized tasks than the other fluid measures. How might these correlations have affected the model outcomes?***

This is exact, and we now acknowledge this possibility in the limitation section which now reads:

…*Second,* *crystallized abilities measures were more interrelated than fluid abilities measures. The rather strong cross-domain associations among crystallized abilities have been documented before and could reflect the combined role of differentially stable personal attributes and environmental developmental building blocks (1,60). Yet, this conceptual/functional divergence may have limited the capacity to center on a core fluid construct, and thus its predictive capacity*.

As suggested by Reviewer 1, we forced a latent "fluid" score in the model for both QNTS and QLSCD. These analyses are now described in a subsection of the new Sensitivity Analyses, and the resulting models presented in Supplementary Figures S5 and S6. Reviewer 1 also proposed that we run a reverse model (one in which Fluid abilities are considered as the mediator), which we did. These “reverse models” are also presented and discussed in this subsection which now reads:

*Examining the putative role of a latent fluid factor. In the proposed model, we derived a latent factor for crystallized abilities, but not for fluid abilities due to their moderate intercorrelation. This means that measures of fluid abilities yielded more measurement error, which could affect their predictive validity. Accordingly, we forced the calculation of a latent fluid factor to evaluate its contribution in the mediational model. The resulting models are presented in supplementary Figures S5 and S6. In both QNTS and QLSCD, the resulting latent fluid factor (1) was biased toward visual-spatial skills (factor loadings of .65 in QNTS, as well as .59 and .57 in QSLCD were all higher than all other loadings), (2) was highly predictive of crystallized abilities (latent score), but (3) with the latter mediating the contribution of the former to school achievement in the early school grades, but not after grade 2. Over and above this strong association with “latent” crystallized abilities, this latent factor also predicted school achievement in grade 3 and grade 4. However, the predictive paths directly leading to school achievement were unstable, as shown by their large confidence interval after bootstrapping. The calculation of such a latent fluid factor also allowed to test an alternate, reverse model, where fluid abilities were posited to mediate crystallized abilities. Supplementary Figures S7 (QNTS) and S8 (QLSCD) illustrate this reverse model. In QNTS, the resulting model was again consistent with the important role of crystallized abilities in predicting early school achievement, and with a delayed prediction of later school achievement by fluid abilities. Indeed, as the putative central mediator, fluid abilities (1) did not significantly predict grade 1 school achievement, (2) only marginally predicted grade 3 school achievement, but (3) significantly predicted grade 4 school achievement (but not grade 6 school achievement). Posited as the initial point in the model, crystallized abilities were substantially predictive of fluid abilities (same as in the original model), but over and above this strong association, it also predicted school achievement in grades 1, but not in grades 3, 4, and 6. The case of QLSCD is clearer: although well predicted by crystallized abilities as the putative mediator, the fluid factor score did not predict school achievement at any time in grade school. In contrast, bypassing fluid abilities, crystallized abilities significantly predicted grade 1, and more marginally grade 2 school achievement, but not later. In other words, the reverse model was not only unlikely on theoretical ground, but also it did not provide a convincing picture of the possible mediating role of fluid abilities.*

8. ***Discussion: although a clear summary is provided of the findings, the authors might also say something about potential alternative explanations of the findings. Specifically concerning training, I wondered whether it is desirable, or even possible, to train such broad skills as crystallized and fluid abilities. See for example: Tricot, A., & Sweller, J. (2014). Domain-specific knowledge and why teaching generic skills does not work. Educational Psychology Review, 26, 265-283*.**

Thank you for raising this point to which we gave great attention in this new version. We now discuss at length the issue the subsection *Implications for prevention and intervention*. We specifically discuss Tricot and Sweller (2014) distinction between domain-general versus domain-specific information and try to bridge these notions with that of our findings regarding fluid and crystallized abilities. We also link this with Bailey et al. (2018) discussion of the disconnect between correlational/longitudinal versus experimental findings.

This new section now reads:

…*This is partly in line with Tricot and Sweller (58) who make the distinction between domain-general information (such as fluid abilities) and biologically secondary, domain-specific knowledge (such as knowledge about numbers and literacy). The former is a form of biologically primary knowledge which is acquired naturally and automatically without instruction, whereas the latter is hardly acquired spontaneously, but can be readily taught, learned and memorized to a point where it provides enough guidelines to achieve in various learning areas (59). They maintain that the goal of any education enterprise should be aimed at fostering domain-specific knowledge rather than improving generic skills.*

*The present findings are partly in sync with this view as they demonstrate that mastering relevant domain-specific knowledge starts in preschool, plays out in the early years of school and then becomes assimilated into stable school achievement. Some have argued that this assimilation process comes with increased expertise, which then facilitates scholastic achievement by providing "internal guidance” in learning (59). From a different angle, others have stressed the role of stable personal and environmental factors to account for this stability in cognitive skills and school achievement (60) (see below for an extended discussion).*

*But at the same time, the present study also strongly suggests that learning these domain-specific knowledge and skills also depends on the initial (preschool) levels of fluid abilities. To the extent that fluid abilities can be improved early on (53), they should be promoted and included as targets of early intervention. The present findings indicate that two aspects should be considered in this endeavor: 1) the capacity for early intervention to enhance fluid abilities, and 2) the provision of learning opportunities (i.e., a stimulating environment) so that these fluid abilities can be “invested” in the mastering of relevant crystallized abilities. Future studies should document the extent to which the predictive association between fluid and crystallized abilities depends (i.e., is moderated by) on learning opportunities and contexts (e.g., the use of quality childcare services). Finally, in addition to early intervention, school-based interventions aimed at improving fluid abilities should also be considered.*

*Still, there are persisting doubts about the long-term impact of these early interventions (61), especially when contrasting correlational-longitudinal work to true experimental evidence, the latter showing only modest if any long-term effect. It has been suggested that some stable personal and environmental factors (e.g., SES) could account for the higher continuity in cognitive and school achievement found in longitudinal non-experimental research (60,62,63). To this point, the mediation model accounted for an impressive portion of the variance controlling for sex and SES variables. The additional specificity analyses indicated that these controls only played a moderate role in the prediction model, and interestingly, more so when predicting preschool crystallized abilities than school achievement in 6^th^ grade. In other words, the resulting model could not be accounted for by these persistent personal (sex) and socio-economic characteristics.*

*Nevertheless, it is still possible that unmeasured personality and genetic factors may be at play (60,64). For instance, previous twin studies have shown that fluid abilities are more likely to be accounted for by genetic sources of influences than crystallized abilities who tend to be more closely associated with environmental sources of influence (14,22,65). To the extent that crystallized abilities are more likely to develop through the common nurturing experiences than fluid abilities, we could expect children who initially lagged in early crystallized abilities (e.g., early numeracy and literacy), but with good fluid skills, to catch up when they are exposed to the school curriculum. This could result in fluid abilities playing a more persistent role than early crystallized abilities in later school achievement. Future studies extending beyond primary school should further investigate this possibility.*

9. ***Minor point: could the constructs measured be mentioned in Table 1, rather than the specific instruments? For the control variables, some information is missing.***

Table 1 has been updated. The specific instruments are listed under the label of their presumed construct. The missing information for control variables has been added.

Responses to the Reviewers’ comments.

Reviewer #1

1. ***Abstract: The sentence “Both fluid and crystallized abilities were found to significantly predict school achievement, but only in the early school years” seems at odds with the paper’s conclusion (p. 10, Conclusion section): “…fluid abilities having a small unique contribution to school achievement in the later grades.”***

We have changed the abstract accordingly. It now reads: *The present study documented in two distinct population-based samples the contribution of preschool fluid and crystallized cognitive abilities to school achievement in primary school and examined the mediating role of crystallized abilities in this sequence of predictive associations. In both samples, participants were assessed on the same cognitive abilities at 63 months (sample 1, n = 1072), and at 41 and 73 months (sample 2, n = 1583), and then with respect to their school achievement from grade 1 (7 years) to grade 6 (12 years). Preschool crystallized abilities were found to play a key role in predicting school achievement. They contributed substantially in the early school years, but more modestly in the later years, due to the strong auto-regression of school achievement. They also mediated the association between fluid abilities and later school achievement in the early grades of school, with the former having modest direct contribution to the latter in the later grades. These findings are discussed regarding their implication for preventive interventions.*

2. ***Introduction: The literature on skill development, school readiness, and later achievement has been somewhat nuanced. Authors’ proposed sequential model would gained in being framed within that more recent literature (see for example: Bailey, D. H., Duncan, G. J., Watts, T., Clements, D. H., & Sarama, J. (2018). Risky business: Correlation and causation in longitudinal studies of skill development. American Psychologist, 73(1), 81).***
3. ***Page 5: The brief mention of general intelligence deserves more elaboration. While the distinction between fluid and crystalized domains is adequately justified, how authors’ theory of fluid and crystallized abilities fit within their theory of general cognitive abilities and interpretation and nature of human intelligence should be clarified. (see: Protzko, J., & Colom, R. (2021). A new beginning of intelligence research. Designing the playground. Intelligence, 87, 101559). Doing so would also strengthen the justification for the study’s mediational hypotheses (pg. 6-7).***

We thank Reviewer 1 for these excellent suggestions. As mentioned in our previous responses, we have now restructured sections of the introduction, as well as sections of the discussion accordingly. Both papers, as well as related ones (e.g., van der Mass et al., 2006) are now integrated in these sections. You will be able to appreciate all the minute changes in the edited manuscript, but the main changes are in the following section of the Introduction, as well as in the extended discussion of the findings (see response to point #8 of the Editor) which refers to these previous papers. Here is the edited section of the introduction.

*However, simply comparing the unique contributions of fluid and crystallized skills to school achievement is not the most informative way to understand the developmental processes involved. Fluid abilities, such as executive functions, short-term memory and cognitive visual-spatial skills, have been associated with crystallized abilities, such as numeracy and literacy (22,23). Given the nature of these abilities, fluid skills likely contribute to the acquisition of crystallized abilities(23,24), which means that the latter could play a mediating role in the contribution of early fluid abilities to later school achievement. This possible sequence, whereby fluid abilities facilitate the acquisition of crystallized abilities, relates to actual views regarding the structure and development of cognitive abilities. Some have indeed suggested that the well-documented interrelation generally found among human cognitive abilities result from mutual interactions between various cognitive processes, starting with the more basic perceptual, memory, and reasoning processes (25,26). Skill building theory (27) also posits that simpler skills lay the ground for the later development of more advanced and sophisticated skills or behavior (28,29). Accordingly, the dynamic association between fluid and crystallized abilities could be seen as a special case of asymmetrical mutualism (26), whereby crystallized abilities stem from the interaction (or investment) of fluid intelligence and experience, ultimately leading to achievement in school.*

4. ***Method: Attrition in school achievement measures is relatively substantial and the authors acknowledged in the Discussion section that missingness might not be random. Documenting with a table the relations between attrition patterns and control variables would be informative. Furthermore, since data is missing on outcome variables, it was not entirely clear how helpful was FIML. Additional analyses could be conducted with listwise-deletion models, as well as with a sample having outcome values present across all time-points. Obtained results should be tabled and compared with FIML ones****.*

The relation between attrition patterns and control variables are now presented in Supplementary Table 2. Table 1 already reported the number of participants for each measure. Regarding to the last point (additional analyses), please see our extensive answer to the editor’s comment #6.

5. **While in the case of crystallized ability, measurement error is addressed by modelling a reflective factor. This was not done for fluid ability specific measures which are thus potentially noisier. Modeling the latter with a reflective factor would achieve three things: 1) alleviate this measurement error potential bias; 2) check for results’ robustness; and 3) bring extra evidence for domain-level effect (fluid ability).**
6. **The possibility of reverse causation was alluded to by the authors in the discussion section. Obtained model fits and pathway estimates should be contrasted with those of alternative models taking fluid abilities (and fluid ability factor) as mediator for crystallized ability(ies) modeled as predictor.**

As indicated in answering the Editor’ s comment #7, we now examine fluid abilities earlier in time in QLSCD (41 months) plus we have performed new analyses positing a latent fluid ability factor as an early predictor and a mediator. Reverse causation is always a possibility, but a more decisive test of reverse causation (still within a correlational design) calls for multiple measures at different times in order to use the temporal decalage to assess direction in prediction, such as in a cross-lagged design (e.g., which is a first step in estimating putative causality). We now acknowledge this as a limitation. Our interpretation of the supplementary analyses is that the calculation of a latent factor for fluid abilities may be overstretching the data. Accordingly, we prefer to stick to the original model, but now present this alternative model in the supplementary analyses designed to test the robustness of the model. See our answer to the Editor’s comment # 7 for more details.

7. **It would also be informative to know the decomposition of fluid abilities effect on school achievement in terms of indirect and direct effects’ relative proportions.**

This information is provided in Tables 3 and 4 as well as in the text of the manuscript.

8. **Following Table 2, page-numbering is off**.

This has been corrected.

9. **Discussion: As the authors address implications for prevention and intervention, it would be important to conduct further analyses controlling for a general ability factor (loading on fluid and crystallized ability indicators). This would also give the opportunity to contrast the stability of the effects of that general factor on school achievement with those of the predictor(s) and mediator(s)**.

The idea of a general ability factor prediction school achievement runs contrary to what we aim to illustrate in the present paper. Nevertheless, we ran such a model and found that the resulting latent factor was poorly defined (only 16% and 22 % of common variance among the various cognitive measures), with crystallized abilities (.66 to .86 QNTS; .60 to .72 QLSCD) clearly having the strongest loadings compared to the fluid abilities (.32 to .60 / .19 to .52). Therefore, such a latent score would be strongly biased toward crystallized abilities (due to their higher intercorrelations). In the two cohorts, this latent general ability score significantly predicted school achievement up to grade 6 (with diminished predictive value over time, as expected). However, it is difficult to interpret the nature of this prediction because of the mixed components of this general ability factor.

10. **Contrary to authors’ claim (p. 8, section Implications for prevention and intervention), whether or not early childhood programs (Head Start or others) have been successful in enhancing fluid and crystallized abilities or later school achievement is not at all settled. Since the authors aim to identifying the nature and timing of elements in school achievement causal chain (and by implication, what could be intervened upon and when), it sems crucial to ascertain effects’s precision and robustness of each measure of interest**.

The point is well taken, and we thank Reviewer 1 for raising it. As indicated previously, we now discuss at great length the disconnect between correlational/longitudinal versus experimental findings. With the additional analyses we believe the manuscript now delivers more compelling evidence of the robustness of the proposed sequential model (and the importance of distinguishing, yet developmentally linking fluid and crystallized abilities), while providing a more nuanced interpretation of the findings. We refer Reviewer 1 to our detailed answer to the Editor’s comment #8.

Reviewer #2

**The manuscript reports on two major longitudinal studies each spanning 7-8 years and each comprising around 1000 participants. The conclusions drawn from the analyses are supported by the data. The data have been appropriately and rigorously analyzed with path analytic modeling techniques implemented in the Mplus program. Some restrictions on data access apply, but data may be obtained by filing a request to access from the Research Unit on Children’s Psychosocial Maladjustment Website. The manuscript is well written in standard English and the presentation is structured in an appropriate manner.**

**This is a very interesting manuscript, which is likely to have major theoretical implications, which in turn can provide a basis for interventions to support development of school achievement. A considerable amount of research has been devoted to investigations of the so called “Investment theory” originally proposed by Raymond Cattell, but it has proven difficult to find strong evidence in support of the theory. However, the present study relies on two well-designed longitudinal studies to show that crystallized abilities mediate the relation between early fluid abilities and later school achievement. More concretely, the two studies show that children with higher fluid abilities are better able to acquire relevant crystallized abilities before school start, such as number or letter knowledge, which makes them prepared to achieve in school. This is the fundamental idea of the “investment” theory and the present study is one of the first to longitudinally document this process from preschool through primary school.

Another important finding is the strong auto-regressive paths of school achievement. School achievement was demonstrated to be highly stable, and half-way into primary school the main predictor of school achievement was prior school achievement. This strong stability of school achievement highlights the importance of early preventive interventions focused on both fluid and crystallized abilities in preschool and the first grades. While most successful early intervention programs have targeted crystallized abilities, the present findings indicate the importance both of the capacity for an early intervention to enhance fluid abilities, and of the provision of learning opportunities so that these fluid abilities can be “invested” in the mastering of relevant crystallized abilities.**

**The manuscript thus has the potential to contribute a stronger theoretical basis for research on the early phase of school achievement, but the powerful longitudinal design and technical skills demonstrated in analyses of data are further reasons for accepting the manuscript for publication.**

We thank Reviewer 2 for his/her positive comments and enthusiastic endorsement of the big picture view suggested by the manuscript.
