# Supplementary material for: A sequential model of the contribution of preschool fluid and crystallized cognitive abilities to later school achievement
Source: PLoS One. 2022 Nov 18;17(11):e0276532. doi: 10.1371/journal.pone.0276532 (PMC9674147; doi:10.1371/journal.pone.0276532)
Supplement: Attachment — Submitted filename: Carpentier et al PloSOne Revision 2 Response to Reviewers.docx [file pone.0276532.s011.docx]

Response to the Editor

1. ***Page 37: Given that at this key point in the discussion the authors are enthusiastic about school-based interventions aimed at improving fluency skills, it would be necessary to base this enthusiasm on at least a couple of recent studies that have achieved this desirable improvement. Using Sternberg's work alone (i.e., Reference #53) is not enough to encourage this perhaps unlikely possibility.***

**Our answer.** We have given the outmost attention to this comment and accordingly, rewrote the most part of the section *Implications for preventive intervention*. We think the section is much clearer and now provides a more complete and nuanced interpretation of the early intervention literature. In doing so, we have integrated relevant studies (e.g., Jaeggi et al., 2008; Bergman et al, 2011) and recent reviews (e.g., Guerin, Wade and Mano, 2021; Kassai et al., 2019; Scionti et al., 2020). Please refer to the track change version of the manuscript to appreciate the changes. The section now reads:

*According to Tricot and Sweller (58) we should distinguish domain-general information, a form of biologically primary knowledge naturally acquired without instruction (such as fluid abilities), from biologically secondary, domain-specific knowledge which can be taught, learned and memorized to a point where it provides enough guidelines to achieve in various learning areas (such as knowledge about numbers and literacy) (59). They maintain that the goal of any education enterprise should be to foster domain-specific knowledge rather than improving generic skills. The present findings are partly in sync with this view as they demonstrate that mastering crystallized abilities (i.e., relevant domain-specific knowledge) starts in preschool, plays out in the early years of school and then becomes assimilated into stable school achievement. This assimilation process could flow naturally as the increased knowledge and expertise provide "internal guidance” in further learning, which is then reflected in scholastic achievement (59). From a different angle, some have raise the possible role of “third variables”, such as stable personal and environmental factors to account for this stability in cognitive skills and school achievement (60) (see below for an extended discussion).*

*But at the same time, the present study also strongly suggests that learning these domain-specific knowledge and skills also depends on the initial (preschool) levels of fluid abilities. From an early prevention perspective and to the extent that they can be improved early on (53), fluid abilities should be considered as targets of early intervention. In light of Cattell’s Investment Theory (23), our findings indicate that two aspects should be considered in this endeavor: 1) the capacity for preschool and school-based interventions to enhance fluid abilities, and 2) the provision of learning opportunities (i.e., a stimulating environment) so that these fluid abilities can be “invested” in the mastering of relevant crystallized abilities. Accordingly, future studies should investigate the extent to which the predictive association between fluid and crystallized abilities depends on (i.e., is moderated by) learning opportunities and contexts (e.g., the use of quality childcare services); in other words, they should document to what extent individual differences in the early, baseline levels of basic fluid cognitive abilities interact with these early learning contexts to shape crystallized abilities.*

*Still, there is some divergence when contrasting correlational-longitudinal work to true experimental evidence. Whereas the former provides significant developmental continuity, there are persisting doubts about the long-term impact of these early interventions (61). Indeed, the jury is still out regarding the extent to which early intervention can foster fluid abilities, and whether these gains can then be translated into increased school readiness and achievement. There is some experimental evidence that fluid abilities, especially fluid reasoning, working memory, and executive functions can be successfully trained (53,62). However, still debated is whether these new and early acquired skills can extend their reach to later functionally related behavior and learning, such as preschool crystallized abilities and school achievement (i.e., far-transfer effects) (63). A recent meta-analysis revealed that reasoning training improves general reasoning ability, but with mixed results regarding a possible transfer to academic performance (far transfer effects) (64). Most of these reviewed studies were conducted among adolescent and adults, with only one randomized controlled study of preschool-age children showing that reasoning training improved fluid intelligence (inductive, deductive, and analogical reasoning abilities), but not working memory (65).*

*Early interventions aimed at improving executive functions among young children are more frequent, but their impact is also limited. In a recent meta-analysis, Kassai and colleagues (63) provided evidence for the effectiveness of training executive functions, but not for the far-transfer of these abilities in related areas. Specifically for young preschool children, Scionti and colleagues (66) also reported an overall significant positive effect of cognitive training on executive functions, this time with some generalization across executive functions, but not to other psychological domains, such as learning prerequisites and behavioral aspects. Thus, although extant meta-analyses show that training executive function is possible, the transfer appears limited to tasks tapping the trained abilities (see also (67) for similar conclusions regarding school-based interventions).*

*This uncoupling of findings between longitudinal and intervention work calls for further inquiry into possible missing links. It has been suggested that some stable personal and environmental factors (e.g., SES) could account for the higher continuity in cognitive and school achievement found in longitudinal non-experimental research (60,68,69). To this point, the mediation model accounted for an impressive portion of the variance over and above sex and SES variables. The additional sensitivity analyses indicated that these controls only played a moderate role in the prediction model, and interestingly, more so when predicting preschool crystallized abilities than school achievement in 6^th^ grade. In other words, the resulting model could not be accounted for by these persistent personal (sex) and socio-economic characteristics.*

*Nevertheless, it is still possible that unmeasured personality and genetic factors may be at play (60,70)…*

Response to Reviewer 1

1. ***I had difficulty connecting Table 3 and 4 with Figure 1 and 2. Specifically, how were the indirect effects calculated with later school achievement outcomes (3rd, 4th, and 6th grade)?
   For example: it seems that Table 3, for 3rd grade school achievement should show a Total Indirect Effect (TIE) of about .24 = (.45 x .64 x .50)+(.45 x .22); for 4th grade a TIE of about .16 = (.45 x .64 x .5 x .54)+(.45 x .64 x .18) + (.45 x .07); etc.
   Perhaps, I am missing something. Greater transparency in how Table 3 and 4 estimates were obtained would be helpful.***

**Our response:** Additional non-significant paths must be included in the total indirect effect calculations. For instance, the indirect effect from Visual-spatial skills to School achievement in grade 3 = .27 = (.45 x .64 x .50) + (.45 x .22) + (.07 x .50; visual-spatial skills -> school achievement grade 1 -> school achievement grade 3).

Similarly for School achievement in grade 4, the indirect effect from Visual-spatial skills to School achievement in grade 4 = .28 = (.45 x .64 x .50 x .54) + (.45 x .64 x .18) + (.45 x .07) + (.07 x .50 x .54; visual-spatial skills -> school achievement grade 1 -> school achievement grade 3 -> school achievement grade 4) + (.07 x .18; visual-spatial skills -> school achievement grade 1 -> school achievement grade 4) + (.45 x .07; visual-spatial skills -> crystallized abilities -> school achievement grade 4) + (.08 x .54; visual-spatial skills -> school achievement grade 3 -> school achievement grade 4).

2. ***The alternative model with fluid abilities modeled as a latent variable (Figure S5 & S6) yielded an indirect effect magnitude on School Achievement 1 on par with total indirect effects from individual fluid ability measures. This might be worth further elaboration in the discussion as it is still unclear whether interventions would be more effective targeting specific fluid abilities or more general influences contributing to that Gf factor, or both (e.g., Protzko, J. (2017), Effects of cognitive training on the structure of intelligence; and Nguyen, T., Duncan, R. J., & Bailey, D. H. (2019). Theoretical and methodological implications of associations between executive function and mathematics in early childhood).***

**Our answer.** We thank Reviewer 1 for pointing this out. We now raise this point at the very end of the discussion as it seems to complement well our discussion of a possible genetic propensity accounting for part of the developmental continuity.

*Nevertheless, it is still possible that unmeasured personality and genetic factors may be at play (60,70). For instance, previous twin studies have shown that fluid abilities are more likely to be accounted for by genetic sources of influences than crystallized abilities who tend to be more closely associated with environmental sources of influence (14,22,71). To the extent that crystallized abilities are more likely than fluid abilities to develop through the nurturing experiences, including family-wide experiences, we could expect children who initially lagged in early crystallized abilities (e.g., early numeracy and literacy), but with good fluid skills, to catch up when they are exposed to the school curriculum. This could result in fluid abilities playing a more persistent role than early crystallized abilities in later school achievement. Future longitudinal studies extending beyond primary school should investigate this possibility. It is interesting to note that when modeled as as a latent variable in supplementary analyses, fluid abilities were as predictive of early school achievement as individual fluid measures. This is consistent with Nguyen, Duncan, and Bailey (72) who found the prediction between executive functions and math achievement to be mostly accounted for a general latent factor of executive function. Such a central role of a higher-order latent score in prediction (versus specific components) is compatible with a possible genetic propensity underlying and accounting for the developmental continuity between fluid cognitive abilities and some core components of crystallized abilities and school achievement. But this is an empirical question for future research.*

3. ***Very minor comment: Arrows of the fluid abilities latent variable (Figure S6) point as if a formative factor was modeled (instead of an expected reflective factor).***

**Our answer:** Thank you for noticing this. We have made the adjustments to Figure S6. Arrows of the fluid abilities latent variable now point in the correct direction (toward the manifest variables and not the reverse).

Response to Reviewer 2

1. ***I noticed a few annoying typos;

   Line 310: Maternal education was aggregated into a 4-point scales (from 1 to 4)
   Change to: Maternal education was aggregated into a 4-point scale (from 1 to 4)

   Line 335: index (CFI), the Tucker-Lewis index (TLI), and the small root-mean-square error of approximation (RMSEA)
   Change to: index (CFI), the Tucker-Lewis index (TLI), and the Root Mean Square Error of Approximation (RMSEA)

   Line 374: as a small root-mean-square error of approximation (RMSEA
   Change to: as a small Root Mean Square Error of Approximation (RMSEA

   Line 403: <.001, respectfully) and then at 73 months (β = .33 and Beta = .09, p. <.001, respectfully)
   Change to: <.001, respectively) and then at 73 months (β = .33 and Beta = .09, p. <.001, respectively)

   Table 3. Direct and Indirect Effects of Fluids Abilities on School Achievement Through Crystallized Abilities and Previous Achievement in the QNTS Sample
   Change to: Direct and Indirect Effects of Fluid Abilities on School Achievement Through Crystallized Abilities and Previous Achievement in the QNTS Sample

   Table 4. Direct and Indirect Effects of Fluids Abilities on School Achievement Through Crystallized Abilities and Previous Achievement in the QLSCD Sample.
   Change to: Table 4. Direct and Indirect Effects of Fluid Abilities on School Achievement Through Crystallized Abilities and Previous Achievement in the QLSCD Sample.

   Line 638: and predictive of standardized national exams at the end of primary school. Fourth, the
   Change to: and predictive of standardized national exams at the end of primary school. Fifth, the***

**Our answer:** We wish to thank the reviewer for taking the time to carefully read the manuscript and for pointing out these typos. We have now corrected them.
